# Editorial: The Design of Molecular Tools in Relation to Prions and Their Biosafety

**DOI:** 10.3389/fbioe.2020.638513

**Published:** 2021-01-20

**Authors:** Maria Lurdes Pinto, Leonor Orge, Maria dos Anjos Pires, Jesús R. Requena

**Affiliations:** ^1^University of Trás-os-Montes and Alto Douro, Vila Real, Portugal; ^2^Instituto Nacional de Investigação Agrária e Veterinária, Oeiras, Portugal; ^3^Departamento de Ciências Veterinárias, Universidade de Trás os Montes e Alto Douro, Vila Real, Portugal; ^4^CIMUS Biomedical Research Institute, University of Santiago de Compostela-IDIS, Santiago, Spain

**Keywords:** prions and related diseases, biosafety, biosecurity, misfolding and aggregation, central nervous system

On the 24th and 25th of October of 2019, the 8th Iberian Prion Meeting took place in the beautiful town of Castelo Branco, in central Portugal. The Iberian Prion Meetings have attracted, since their first edition, prion researchers not just from the Iberian Peninsula but from all over Europe and beyond. Much smaller than their “bigger sisters,” the Annual International Prion Meetings, they never aimed at counter-programming them, much to the contrary, they have been a complement to them, focused on fostering regional collaborations and providing a valuable learning experience for novice prion researchers. In the last edition, the organizers coordinated with Frontiers in Bioengeneering and Biotechnology to publish a special issue that, originating from the communications to the meeting, would stretch beyond them to present a view of the state of the art in “the design of molecular tools in relation to prions and their biosafety.”

Little we knew 1 year ago that our reflections on a zoonotic agent would have to be conducted under the shadow of another one. SARS-CoV-2 has changed everything. The Iberian Prion and International Prion meetings of 2020, that should have been held in Corunna and Goettingen, respectively, have been canceled, and it is unlikely that they will take place in 2021, either. During the last Iberian Prion Meeting we discussed the potential dangers of a new prion zoonosis in the wake of the first cases of Chronic Wasting Disease (CWD) in Scandinavia (Benestad et al., 2016; Koutsoumanis et al., 2019), keeping in mind that Castelo Branco is located near the Serra da Estrela, home of the largest population of deer in Portugal. CWD does not seem to be transmissible to humans, but what if it is passaged to sheep or cattle sharing grazing lands with the deer? Does this context sound familiar? Bat coronaviruses passaged to dromedaries, pangolins or civets…Bovine spongiform encephalopathy (BSE) prions, HIV, Ebola, coronaviruses…all the major health crises of the last decades are zoonoses. We therefore have to maintain research on all these agents, because it is not “if” but “when” new outbreaks, perhaps of more infectious and/or more deadly versions of them will strike.

In this special issue of Frontiers in Bioengeneering and Biotechnology, 11 articles from 83 authors describe and explore an array of tools to identify, diagnose, study and fight prions. Unfortunately, diagnosis of prion diseases is often done post mortem, impairing the management of disease in the individual and preventing early screening. Early detection methods are thus of pivotal importance in this research field. Two articles (Zerr et al.; Pires Barbosa et al.) focus wholly and one (Ascari et al.) partially on the Second-Generation real-time quaking-induced conversion (RT-QuIC), a technique that is revolutionizing diagnostic of prion diseases. Recent advances in its optimization and application to cerebrospinal fluid are presented and discussed. Additional ante-mortem diagnostic methods are reviewed by Ascari et al. and Konold et al. reveal the importance to reassure the current tools for TSE screening in animals, namely in asymptomatic goats. They point out to the need of reviewing current European surveillance regulations for scrapie screening, by testing other tissues than central nervous tissue, in order to keep an accurate surveillance and control of animal prion diseases. The article by Konold et al. sheds also light on the pathology of prion diseases, a subject particularly addressed by López-Pérez et al. In their article, regarding autophagy, the authors review the data from *in vivo* and *in vitro* studies that suggest a decrease of autophagic activity or a deterioration of the lysosomal degradation process in prion diseases. Whether these features are a prerequisite or consequence of prion-induced toxicity still remains unclear. del Río and Ferrer report on cutting edge technology to study neurodegenerative and metabolic diseases, in which the accumulation of misfolded proteins are a key-factor. They explain that microfluidics and lab-on-chip platforms may be valuable methods to improve understanding of the seeding and spreading processes of these “prion-like” amyloids and constitute an important alternative to unnecessary animal experimentation following the 3Rs of biological research. A *sine qua non* step on the developing of the disease is prion replication, and in an original research article, Spagnolli et al. describe the use of computational methods to address the molecular underpinnings of the heretofore enigmatic “deformed templating” conversion of different propagative PrP conformers. Also taking advantage of a biotechnological approach applied to the study of basic aspects of prion structural biology, Silva et al. describe the design of extremely sensitive, quantitative nanoLC mass-spectrometry methods to probe the structure of PrP^Sc^. By treating PrP^Sc^ with lysine-specific chemical probes and measuring the extent of modification of each individual one, they were able to scan the correlation of each one of them with abrogation of infectivity. Only one K_220_ had a reactivity that is consistent with the loss of infectivity, which the authors discuss in the context of recent PrP^Sc^ models.

While understanding prion propagation is of an intrinsic biological interest, of course the ultimate goal of prion biology and biotechnology is to develop therapies for prion diseases. Peña-Díaz et al. describe the use of a High Throughput Screening protocol based on Thioflavin-T fluorescence, light-scattering measurements and Transmission Electron Microscopy. This pipeline was used by the authors to discover and characterize a small molecule that acts as potent inhibitors of α-Synuclein amyloid formation. This article, besides showcasing the application of bioengeneering approaches to the search of prion therapies, highlights the interconnection of prion diseases with other protein misfolding-related (prion, prion-like of prionoid…) diseases.

Regarding prion biosafety and biosecurity, Eraña et al. used a method based on cyclic amplification of a starting PrP^Sc^ pool of molecules, protein misfolding shaking amplification (PMSA) to develop a sensitive and ingenious method to evaluate prion decontamination procedures. Moudjou et al. also present new data on the application of PrP^Sc^ amplification techniques, RT-QuIC (again…) and protein misfolding cyclic amplification (PMCA) to solve the problem of procedures for decontamination of surgical instruments.

As guest editors of this special issue of Frontiers in Bioengeneering and Biotechnology, we hope that readers will enjoy this collection of articles that highlight the importance of technological advances, from computational methods to mass spectrometry to microfluidics in the quest to understand, detect and decontaminate prions. However, as investigators search at the molecular level, we must never forget the great importance of the broader relationships occurring at the interfaces of humans, animals, and the environment in the pursuit of global health.

## Author Contributions

All authors listed have made a substantial, direct and intellectual contribution to the work, and approved it for publication.

## Conflict of Interest

The authors declare that the research was conducted in the absence of any commercial or financial relationships that could be construed as a potential conflict of interest.
